# A Simple In Vitro Method to Determine Bactericidal Activity Against *Mycobacterium abscessus* Under Hypoxic Conditions

**DOI:** 10.3390/antibiotics14030299

**Published:** 2025-03-13

**Authors:** Ruth Feilcke, Robert Eckenstaler, Markus Lang, Adrian Richter, Peter Imming

**Affiliations:** Institut für Pharmazie, Martin-Luther-Universität Halle-Wittenberg, Wolfgang-Langenbeck-Straße 4, 06120 Halle, Germany

**Keywords:** hypoxia, low-oxygen persister, MBC determination, *Mycobacterium abscessus*, non-replicating persister, phenotypical resistance

## Abstract

**Background/Objectives**: Non-replicating persisters (NRPs) of *Mycobacterium abscessus* are a bacterial subpopulation that can survive in the host under unfavorable conditions, such as hypoxia or nutrient starvation. The eradication of these bacteria is difficult, which is one reason for the long treatment duration and treatment failure. The drug discovery process should therefore contain methods to screen activity against NRPs. **Methods**: A hypoxic environment is used to generate NRPs of *M. abscessus* that are termed low-oxygen persisters (LOPs). For this, an oxidation process is used to transition a replicating culture of *M. abscessus* distributed in microtiter plates within a sealable box into LOPs. Colony counting, automated object counting, bactericidal activity determination of known agents, and confocal laser scanning microscopy are used to study the obtained culture. **Results**: The obtained culture shows typical attributes of non-replicating cells, such as significantly reduced replication, the reversibility of the LOP state under aerobic conditions, delayed regrowth on solid medium, altered morphological patterns on a single-cell level, and phenotypical resistance against a variety of clinically relevant antimycobacterial compounds. The study reveals metronidazole and niclosamide as bactericidal against *M. abscessus* LOPs. These compounds can be used as LOP verification compounds within the described model. **Conclusions**: Our model is easily implemented and quickly identifies compounds that are inactive under hypoxic conditions. It can therefore accelerate the identification of clinically effective antimycobacterial drug substances, and can be a helpful tool during the drug development process.

## 1. Introduction

Non-tuberculous mycobacteria (NTM) are a group of mycobacteria distinct from *Mycobacterium tuberculosis* and *Mycobacterium leprae*, the causative agents of tuberculosis (TB) and leprosy, respectively. As NTM infection rates are rising globally, they are becoming a concern for many health systems worldwide [1,2]. The reported treatment successes are “similar to those for extensively drug resistant tuberculosis”, in the region of 54% for *M. abscessus* subsp. *massiliense* and 33% for *M. abscessus* subsp. *abscessus* [3]. Among NTM, the fast-growing *M. abscessus* is intrinsically insensitive to many known TB drugs and most classes of antibiotics [4]. In the last 20 years, tremendous effort to develop new TB drugs has led to three newly approved drugs and a robust drug pipeline [2]. In contrast, the drug pipeline for NTM and *M. abscessus* in particular is sparse [2,5], with not a single new compound entering clinical trials while pre-clinical development is gaining momentum [6].

In addition to the lack of effective treatments for *M. abscessus* disease, another key challenge is the existence of a subpopulation of non-replicating persisters (NRPs) during an infection. NRPs are substantially more difficult to eradicate, and contribute to the characteristically long treatment regimens required for infections with *M. abscessus* and other pathogenic mycobacteria. *M. abscessus* is an obligate aerobic bacterium, which easily adapts to microaerophilic conditions through metabolic reprogramming, leading to a larger subpopulation of NRPs that are able to survive even under hypoxia. In hosts, such low-oxygen and hypoxic conditions are found in granulomas in the lung [7,8] and in mucous plaques of cystic fibrosis patients’ airways [9]. Once *M. abscessus* has entered a non-replicating state, most of the few effective drugs become inactive [10]. Among the different methods used to screen against NRPs, including acidic stress [11,12], carbon starvation [13,14,15], nitric oxide stress [16] and potassium starvation [17,18], hypoxic models [19,20] are the most challenging ones. Most of the screening methods have been developed for *M. tuberculosis* NRPs. Only some of them have been transferred to *M. abscessus*. However, existing methods mimicking hypoxic conditions for *M. abscessus* are either laborious and not microtiter plate-compatible [10,19,21,22], or rely on genetically modified strains [20,23] or the availability of technical devices that ensure hypoxic conditions, like an Anoxomat or a chemostat [12,20,23].

To fill this gap, we sought to develop a hypoxic model that enabled the efficient screening of compounds in a simple way. To emphasize the fact that non-replicating persistent cells in our set up result from their survival in an environment with low levels of oxygen, we refer to these *M. abscessus* cells as low-oxygen persisters (LOPs). The assay set up to investigate compound activity against LOPs should convert an aerobic *M. abscessus* culture to an LOP culture by use of readily available components. The LOP culture so obtained is expected to exhibit typical characteristics of non-replicating persisters, such as markedly less replication, the reversibility of the NRP state (known as resuscitation [18]), morphological changes and phenotypical drug resistance [24]. We confirmed these properties by investigating the growth behavior of the culture during and after hypoxic conditions, using single cell microscopy to determine the morphological appearance of the cells and measuring the activity of various clinically relevant antimycobacterial compounds.

## 2. Results and Discussion

### 2.1. Generation of a Hypoxic Environment by Activated Iron Wool

**Figure 1:**
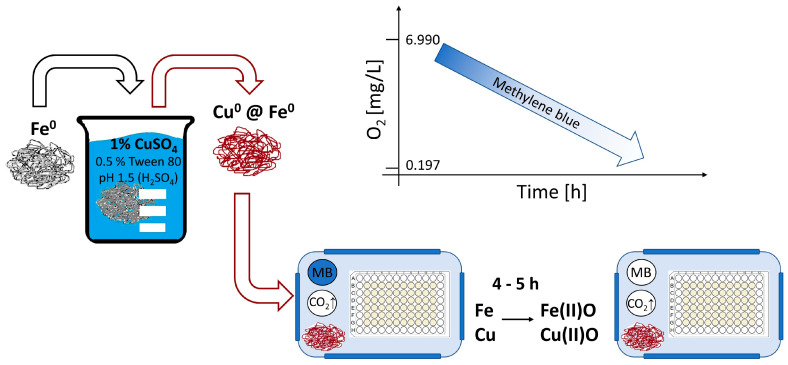
Schematic presentation of procedure followed to generate a hypoxic environment (approach adjusted from [25]). Activating of iron wool grade 0 by dipping into an acidic solution of copper sulfate and polysorbate 80 (Tween 80) to spot the iron wool with metallic copper. The activated iron wool is then placed in a sealed box together with a microtiter plate containing a liquid culture of *M. abscessus*. The rapid oxidation of iron and copper leads to the depletion of oxygen concentration to achieve hypoxic conditions within four to five hours. The concentration of oxygen can be monitored with a solution of methylene blue (MB) that changes from blue to colorless upon hypoxia. The reported oxygen levels within the graph correspond to dissolved oxygen within the bacterial suspension under aerobic and hypoxic conditions, respectively.

Oxygen depletion in the LOPs assay was achieved by the oxidation of activated iron wool in a sealable box, monitored with a solution of methylene blue (MB) that changed from blue to colorless. All the components were placed next to the bacterial culture in an airtight box, as shown in Figure 1. This approach was first described by Parker [25], and has not been applied to mycobacteria. The fading of the indicator MB began approximately 4 h after the box was closed, and the decolorization took place about one hour later. We measured the concentration of oxygen after the decolorization of MB, and found 4.5 to 7.4 vol % of oxygen in the headspace and 1.29% air saturation of oxygen within the bacterial suspension, corresponding to 0.197 mg/L or 0.27% dissolved oxygen (see Supplementary Materials Figure S3).

Parker’s method met the requirements of our project to establish a robust method with easily accessible components in order to simplify the creation of a hypoxic environment. Most authors reporting on mycobacteria under hypoxic conditions did not quantify the amount of oxygen [10,21,22]. Instead, they also relied on MB as an indicator to monitor the oxygen content. The MB turned colorless in our setup, and the levels of oxygen achieved within the media were in the range of those under Wayne’s model [19]. Although MB is a common indicator used to control hypoxic conditions, its application to the bacterial suspension will result in some (unspecific) interference due to the oxidizing potential of the substance. This could include reactions with components of the culture medium or redox active co-enzymes within the respiratory chain of the bacteria, or the direct inhibition of enzymes, as shown for other species. Keeping the indicator in a separate vial within the setup avoids these disadvantages. Parker’s method avoids a sudden onset of hypoxia, as has been described as sterilizing for *M. tuberculosis* [26] and *M. abscessus* [23]. The sachets reduce the amount of oxygen to below 1% within 30 min—too fast for mycobacteria to adapt to [23,26]. Oxygen removal by Parker’s method is fast enough to largely avoid the resuscitation of non-replicating cultures after oxygen re-entry during compound addition (see section on evidence for *M. abscessus* transitioning to the LOP state). This eliminates the need to use anaerobic tents or glove boxes to add compounds, again simplifying the procedure and making it more cost-effective.

### 2.2. M. abscessus LOP Assay for Determination of Drug Activity In Vitro

The method described above was used to generate *M. abscessus* LOPs directly from a replicating culture of *M. abscessus* via incubation in an airtight box under hypoxic conditions.

**Figure 2:**
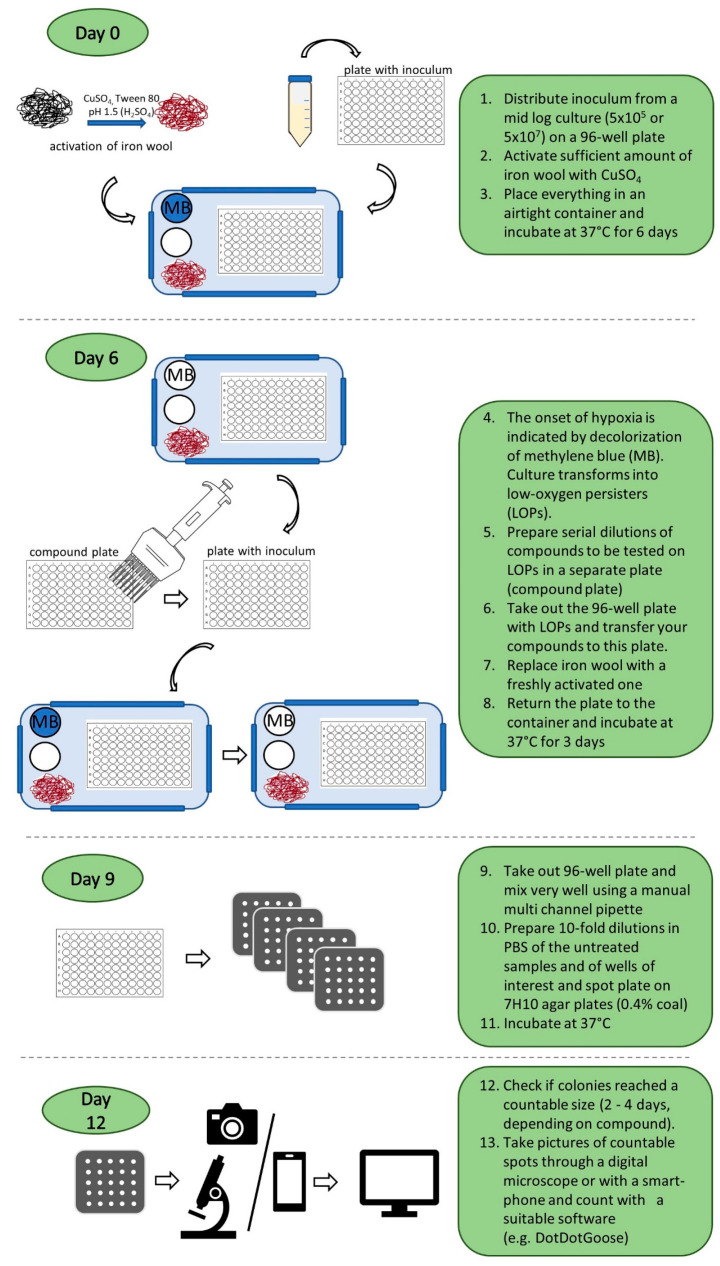
Detailed description of the assay procedure to determine bactericidal activity on *M. abscessus* LOPs. MB: methylene blue solution used as an indicator of oxygen concentration. White circle: saturated carbonate solution for the release of CO_2_.

Figure 2 depicts the procedure of the assay, which was performed in the same microtiter plate as was used for culture transitioning. The activated iron wool ensured a rapid onset of hypoxic conditions after the re-entry of oxygen due to the addition of test compounds. A robust dilution process in microtiter plates with subsequent spot plating on agar with counting software precluded tedious manual colony counting. Details of the method are given in the Methods section.

Preliminary experiments revealed that the red fluorescence protein (RFP)-harboring strain of the rough morphotype of *M. abscessus* (pTEC27) [27] was unsuitable for culturing under hypoxic conditions due to the extensive loss of the functional plasmid, even though hygromycin selective culturing was employed. We suppose that stressful conditions can lead to antibiotic resistance being incorporated into the bacterial genome, while extrinsic, useless genetic material is eliminated.

Wayne’s model is normally applied for the determination of in vitro compound activity against hypoxic mycobacteria. It requires one tube for each compound concentration. In contrast, the LOP assay reported here is performed in microtiter plates, enabling higher throughput. Wayne’s model relies on the consumption of oxygen by bacteria to deplete oxygen in the set up. In our model, the depletion of oxygen is based on a quick oxidation process that enables the addition of compounds to the hypoxic culture without needles for safer handling (BSL 2). Our approach to a hypoxic model is a less complex method compared to the LORA procedure [20], wherein a hypoxic parent culture needs to be prepared prior to the actual assay with Wayne’s model. The maintenance of a hypoxic parent culture to ensure comparable conditions over several experiments is not necessary in the LOPs assay. Our method includes CFU counting, which can be tedious, and this is why we plated spots of several dilutions simultaneously [23] instead of preparing spread plates from bacterial suspensions, where one agar plate is necessary for every single dilution. This approach enabled us to determine the activities of twelve compounds at different concentrations within one day. Spreading out dilutes the remaining antibiotic compound to a greater extent than spot plating. Therefore, the inhibition of bacterial growth on solid medium is possible. To exclude compound carry-over, 0.4% active carbon was added to the solid medium. In doing so, colonies could be detected even in undiluted samples of high concentrations of antimycobacterial compounds. The use of a digital camera in combination with counting software further accelerated the process. This approach is also transferable to smartphone camera systems, precluding additional costs for microscopes, which is in accordance with the goal of making this method available at low costs.

### 2.3. Evidence of M. abscessus Transitioning to the LOP State

#### 2.3.1. Activity of Nitroaromatics Against *M. abscessus* LOPs

To ensure the LOP state of the culture in the set-up, an LOPs verification compound for *M. abscessus* was needed. This compound should ideally be inactive against *M. abscessus* under aerobic conditions, but should become bactericidal under hypoxic conditions. The approach of a “switch compound” was inspired by Cho et al., who successfully applied it to *M. tuberculosis* in the LORA [20]. From among the FDA-approved drugs, two substances were chosen, as follows: metronidazole, because of the long ongoing discussion about its activity against NRPs of *M. tuberculosis* (for a summary, see reference [12]), and niclosamide, due to its activity against nutrient-starved *M. abscessus* cells, another type of NRP [15]. To the best of our knowledge, for both compounds, their dose-dependent activities under hypoxic conditions on *M. abscessus* have not been investigated. First, MICs for both compounds were determined under aerobic conditions using the broth dilution method, and we did not find any growth inhibition up to 100 µM for metronidazole, or any growth inhibition of at least 90% up to 50 µM (due to solubility issues) for niclosamide.

**Figure 3:**
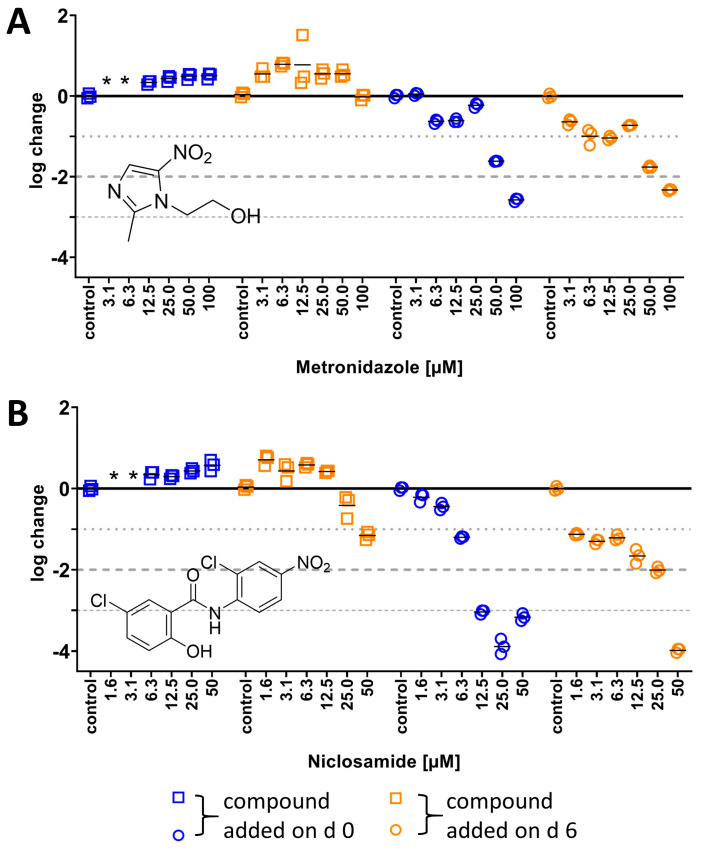
Time- and dose-dependent logarithmic changes in the CFU/mL of (**A**) metronidazole and (**B**) niclosamide on *M. abscessus* LOPs. **Blue**—drug was added on day 0 of experiment, **orange**—drug was added on day 6; **left, squares**—read out on day 3, **right, circles**—read out on day 6. For the calculation of log change, see the Materials and Methods section. The experiment was carried out in triplicate and is representative of two independent experiments; * not determined.

Bactericidal activity under hypoxic conditions was then investigated at two different points in time for drug addition. By following our protocol and adding compounds on day 6, the activity on an LOP culture was determined (Figure 3, orange data points). Additionally, the activities of both compounds were investigated by following a slightly different procedure, adding compounds on day 0 of the experiment, and thereby exploring activity on a culture that transitions into an LOP in the presence of the compound (Figure 3, blue data points). For both compounds, time-dependent activity under hypoxic conditions was found. Metronidazole (Figure 3A) and niclosamide (Figure 3B) reduced CFUs after six days to a greater extent than after three days.

For metronidazole, the dose-dependent activity was independent of the time it was added to the bacterial suspension. The activity on an LOP culture (blue data points, Figure 3A) as well as on a culture that transitioned into LOPs in the presence of metronidazole (orange data points, Figure 3A) was similar when comparing data from the same point in time—either no activity after three days, or a reduction in CFUs by >2 log units for 100 µM in both cases. This suggests that the bactericidal quality under hypoxic conditions was only time-dependent at higher concentrations.

The bactericidality of niclosamide (Figure 3B) appeared to be influenced by the metabolic state of the culture, since there were differences in colony reduction between LOP cultures (orange data points, Figure 3B) and cultures that transitioned into LOPs in the presence of niclosamide (blue data points, Figure 3B). No reduction was observed after three days when 50 µM niclosamide was added on day 0. In contrast, CFUs were reduced within three days by 1 log unit when compounds were added on day 6. After six days of incubation, niclosamide was more active when added on day 6 (MBC_90_ 1.6 µM) compared to addition on day 3 (6.3 µM). However, similar values were observed at higher concentrations independent of when it was added.

The MICs for metronidazole and niclosamide were consistent with those in the literature, where no inhibition was found for metronidazole and MIC_90_ values for niclosamide were reported to be >50 µM [28,29]. However, investigations in the literature on hypoxic *M. abscessus* were carried out without verification compounds [21,22,23]. In most studies, methylene blue served as the sole control. This indicator only allows for monitoring of oxygen concentrations, but does not prove the transition of replicating *M. abscessus* to LOP cells. In contrast, our results reveal two FDA-approved drugs to support the formation of *M. abscessus* LOPs. There is little information on the activity of niclosamide against *M. abscessus* under hypoxic conditions to compare our results with. Lanni et al. included this drug at its maximum serum concentration (1.8 µM [22,30]) in their study. This concentration was in the range of the lowest concentration investigated in this study. They described niclosamide as bacteriostatic under aerobic conditions on *M. abscessus,* but reported only minor differences in CFU/mL when comparing untreated hypoxic *M. abscessus* with treated cultures. Regarding the differences in their set-up (they employed the Wayne model and a clinical isolate instead of the strain ATCC 19977), we consider the observed 1-log reduction at 1.6 µM in our study to be comparable to their results. Nutrient-starved *M. abscessus* was reported to be more susceptible to niclosamide, with a 2-log reduction after 3 days at 25 µM [15]. *M. abscessus* LOPs showed a 0.5-log reduction only after 3 days at 25 µM in our study. The results suggest that instead of niclosamide, metronidazole can be used as a verification compound at 100 µM, leading to a >2-log reduction in CFUs. In the LORA, the lowest concentration of metronidazole used to verify the non-replicating state was 100 µM [20]. This is still 50 times less than what was used by Khan et al. [31] as a control in a hypoxic *M. tuberculosis* model. While a concentration of 100 µM and higher is too high to consider a compound as active against mycobacteria [32], this is acceptable for the present sole purpose of culture transition verification. To simplify the procedure, either 100 µM metronidazole or 50 µM niclosamide were added on day 0 of the assay. Thereby, at least a 2-log reduction in CFUs was observed and served as proof that the culture transitioned from replicating to *M. abscessus* LOPs.

#### 2.3.2. Growth Behaviour of *M. abscessus* LOPs

If the bacterial culture transitions to LOPs, the growth behavior should be seen to differ from a regular bacterial growth curve, as LOPs belonging to NRP should replicate significantly slower [24]. The absence of turbidity was a first indicator that *M. abscessus* did not divide as usual under these conditions within three or even six days. In this time range, *M. abscessus*, when incubated under aerobic conditions, typically reached OD values of about 0.4 and higher, so turbidity would be easily detectable. CFU counting on 7H10 agar developed a comparable number of colonies as were seen for the respective inoculum. This supports our hypothesis of slower replication within our setup.

**Figure 4:**
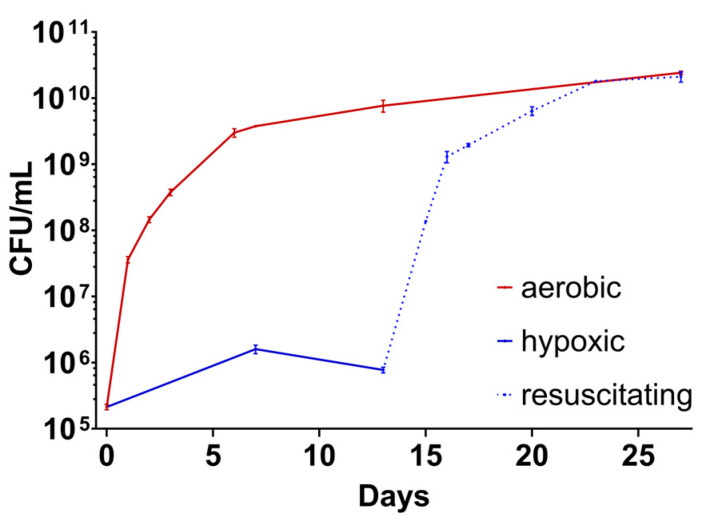
Growth of *M. abscessus* culture. **Red**: incubation in a carbon dioxide incubator (5%). **Solid blue**: incubation in the hypoxic box. **Dashed blue**: resuscitating from hypoxia (5% CO_2_). Hypoxia was ended on day 13 of this experiment by opening the hypoxic box and transferring the 96-well plate to the same incubator as the culture denoted by the red curve. Data points are the mean of three replicates, and the experiment shows typical growth curves derived from two independent experiments.

To further investigate the difference between the starting inoculum and the number of bacteria after being subjected to hypoxia, as well as to ensure viability and cultivability on solid medium, the difference in CFU numbers was investigated over time under aerobic and hypoxic conditions for *M. abscessus*. For this, a culture of *M. abscessus* in the exponential growth phase was distributed across two different 96-well plates. One plate was incubated in the hypoxic box and one plate under aerobic conditions (5% CO_2_). At defined points in time, samples were taken, and the CFU/mL was determined by plating on agar. Cultures in both microtiter plates were analyzed over the same period of time. For the hypoxic plate, fewer data points were collected (blue solid line, Figure 4). As the atmosphere in the hypoxic chamber is affected every time it is opened, we sampled every time the box needed to be opened. After 13 days, the longest period of hypoxia investigated, the plate incubated in the hypoxic box was exposed to aerobic conditions to enable recovery from hypoxic stress. This process was followed by CFU number evaluation (blue dashed line, Figure 4).

Figure 4 shows the value of CFU/mL over the duration of the experiment. The red curve displays the *M. abscessus* culture that was grown under aerobic (with 5% CO_2_) conditions. The curve shows the expected growth behavior of a culture during an exponential growth phase followed by a stationary phase. The lag phase, which precedes the exponential phase, is skipped due to inoculation from a log phase culture to obtain about 5 × 10^5^ CFU/mL. The growth behavior of the hypoxic culture is shown in solid blue. The displayed values represent the amount of CFU/mL that is obtained by the setup in our assay over 13 days. When cultured under hypoxic conditions, there was little change in the number of CFU/mL, which is due to the lower oxygen content that led to a reduced division rate of the bacteria (×10 (hypoxic) vs. ×10^4^ (aerobic) within 7 days). An increase in CFU/mL was detectable at day 7, followed by a slight decrease until day 13. The data reflected by the dashed blue line represent the CFU numbers of the culture analyzed during the resuscitation phase in 5% CO_2_ after hypoxic incubation for 13 days. Resuscitating cells showed similar growth behaviors as unstressed cultures (compare the red solid and blue dashed lines, Figure 4).

Given the low scatter of values and the typical growth curve as observed under aerobic conditions (see Figure 4), plating from 96-well plates seemed to give reliable results, and plating from cultures grown in tubes appeared to be unnecessary. This enabled the use of microtiter plates in our setup. Manual mixing for at least 20 cycles and the use of polysorbate 80 were useful tools to avoid the clumping of bacteria, and we yielded reasonable results with this approach. We further concluded from the CFU numbers that the cells were still viable and cultivable, which suggests that the depletion of oxygen with activated iron wool was slow enough to not sterilize the culture. The differences in CFU numbers under hypoxic conditions might be explained via the oxygen available at the beginning of the experiment. Because of the slow onset of hypoxia, which is essential to avoid the sterilization of the culture [23,26], the conditions were growth-supporting in the beginning of the assay, and the cells were able to replicate. This led to a slight increase in CFU/mL comparable to the conditions in the Wayne model, whereby cells consumed the remaining oxygen by multiplying. After the onset of hypoxia (decolorization of MB after about 5 h), *M. abscessus* survived as LOPs, and the cell numbers remained at a similar level. During up to 13 days of hypoxia, the number of CFUs slightly decreased. This could be due to the transition to viable but non-cultivable cells [33], or to some cells entering a non-viable state, as observed by Lanni et al. from day 10 onwards [22]. When comparing growth rates during the first 2 days of growth in aerobic and recovering cultures (Figure 4), no significant differences (*p* = 0.325) between the two conditions were found. This indicates complete resuscitation in liquid medium after two days.

#### 2.3.3. Resuscitation of *M. abscessus* LOPs on Solid and in Liquid Medium

**Figure 5:**
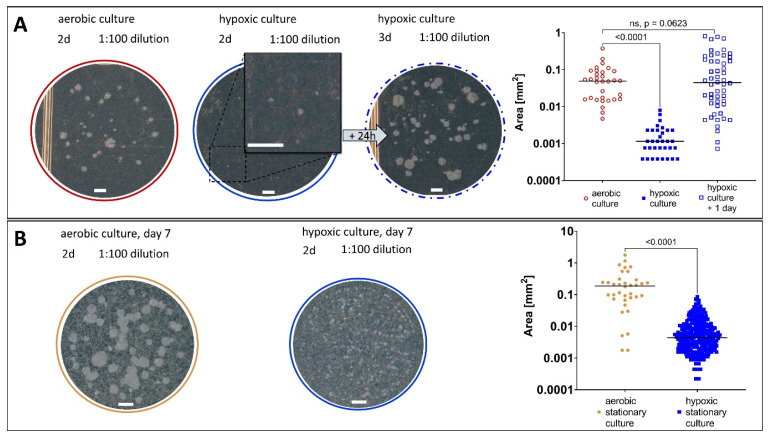
Sizes of *M. abscessus* colonies on solid medium (7H10, ADS, Glycerol) under different conditions. The plates were incubated in 5% CO_2_ at 37 °C. The areas of detected colonies are displayed with their respective medians, and were compared by t-test within one experiment. (**A**) Aerobic culture (red) in comparison to a culture after 7 days of hypoxia (blue). After another 24 h, the colonies coming from a hypoxic culture reached a countable size as well (blue dashed). (**B**) The colonies of *M. abscessus* from an aerobic culture in stationary phase (day 7 of experiment, golden) and colonies from a culture after 7 days of hypoxia (blue), scale bars 1 mm.

Comparing the sizes of colonies on solid medium after the same incubation time, colonies derived from LOP cultures (Figure 5A, blue) were found to be smaller than those from regular *M. abscessus* cultures (*p* < 0.0001; Figure 5A, red).

This delayed growth was compensated for by an extended incubation time (after an additional 24 h, there was no significant difference in the area of colonies compared to the aerobic culture, *p* = 0.063; Figure 5A; dashed blue). The additional 24 h are a suggested time frame for the resuscitation on solid medium of LOP cells. To exclude the impact of the bacterial metabolism in the stationary phase on the colony size, the size of colonies of an *M. abscessus* culture that had already entered the stationary phase (day 7 of experiment, Figure 5B, red) was compared with that of colonies derived from hypoxic conditions (Figure 5B, blue), and we found the latter to be significantly smaller (*p* < 0.0001). The recovery from stress due to the stationary phase (less nutrition due to many cells) seemed to occur faster than recovery from the LOP state due to hypoxic stress. That stationary cultures are indeed different from hypoxic cultures was described for *M. tuberculosis* by Voskuil et al., by studying their differential gene expressions [34].

The delayed time to evaluate CFUs is a typical attribute of *M. tuberculosis* NRP, and this property was used as a control by Cho et al. to verify the transition of *M. tuberculosis* into NRPs in their hypoxic model [20]. For *M. abscessus*, it was necessary in our experiment to increase the incubation time of the agar plates by a factor of 1.5 (24 h). Compared to data for *M. tuberculosis*, which is a slow-growing mycobacterium, the delayed growth seems to indicate a reduced metabolism [35,36].

**Figure 6:**
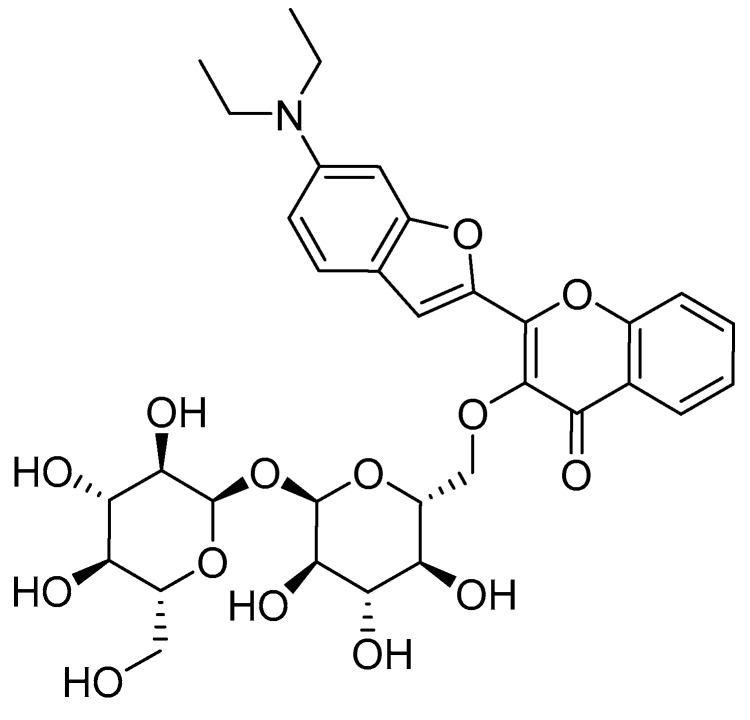
Structure of the 3-hydroxychromone dye (compound **1**) used to study regrowth in fresh liquid medium of *M. abscessus* cultures derived under different conditions.

We further proved that the delayed regrowth of *M. abscessus* LOPs also occurred in liquid medium using a method previously published by our group [37] that uses a fluorescent 3-hydroxychromone dye conjugated to trehalose (compound **1,** Figure 6) [38] in combination with automated object counting for the detection and evaluation of mycobacteria.

**Figure 7:**
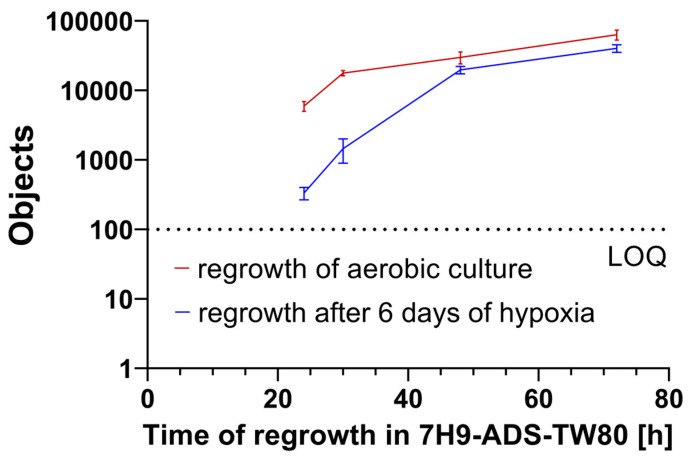
Regrowth of *M. abscessus* in liquid medium, starting from the same inoculum for (red) aerobic and (blue) hypoxic conditions (values at t = 0 h were below LOQ, and are therefore not displayed within the graph.) LOQ, limit of quantification.

As in the growth experiment on solid medium, here, a culture was pre-treated under either hypoxic or aerobic conditions. Then, the cultures were diluted in fresh culture media, sampled, stained with **1** and analyzed by automated object counting (see Section 3.10). A significant difference in the object counts for both cultures at several sampling points was found (see Figure 7), suggesting that the *M. abscessus* LOPs culture indeed showed a delayed re-growth compared to aerated cultures. This result is consistent with the delayed growth rate on solid medium, and indicates a process of resuscitation in which the reduced metabolism of bacterial cells takes time to be reactivated, which slows down the growth rate of the culture.

The absence of an exponential growth rate in liquid medium under hypoxic conditions, the different sizes of colonies between the two cultures on solid medium, as well as the delayed re-growth in liquid medium all strengthen our hypothesis that cells subjected to this setup are in an inactive state. This corroborates that cells in our set up transitioned to LOPs. The ability to recover from hypoxic stress and to transition from LOPs back to actively replicating cells is consistent with the observation made for *M. tuberculosis* in the literature [39], and our results demonstrate that this applies to *M. abscessus* LOPs on solid as well as in liquid medium.

#### 2.3.4. Morphology of *M. abscessus* Single Cells

**Figure 8:**
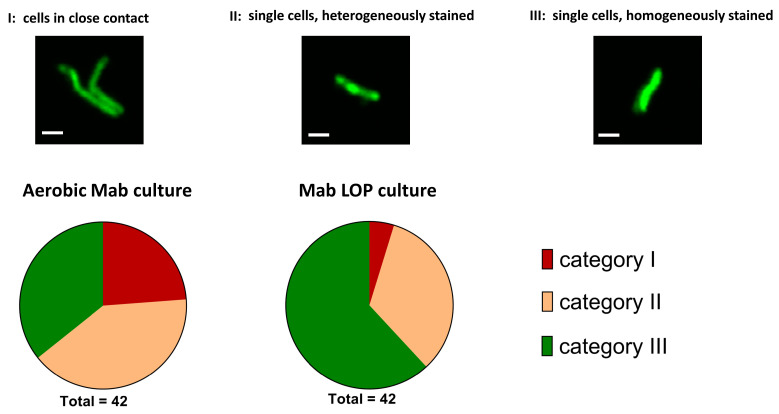
**Upper part**—Exemplary pictures of the three categories; I cells in close contact to each other, II single cells, heterogeneously stained with brighter staining at the septum and poles compared to other parts of the cells, III single cells, homogeneously stained. Scale bar: 1 µm. **Lower part**—Proportion of cells in the different categories in aerobic and hypoxic bacterial populations; Mab *M. abscessus*.

Cells were stained with compound **1** to investigate the morphology of *M. abscessus* single cells by confocal laser scanning microscopy. A series of pictures of a representative selection of *M. abscessus* cells was taken for each population. The study was performed as a single blinded investigation, with the person selecting about 40 cells per population while not knowing from which culture the cells were taken. The cells of a culture were sorted by their morphological appearance into three categories before unblinding the study. Figure 8 shows the percentages of different morphological patterns in the total populations of aerobic *M. abscessus* and *M. abscessus* LOPs. All pictures are shown in the Supplementary Materials (Figure S4). Category I includes cells that appear close to one another, category II includes single cells that show brighter staining at the septum and poles compared to other parts of the cell, and category III includes single cells that are homogeneously stained throughout the cell. The aerobe culture was almost evenly distributed over the three categories, with most pictures placed in category II (17/42), followed by category III (15/42) and category I (10/42). In the hypoxic culture, more than half of all pictures were attributed to category III (26/42), followed by category II (14/42), with only two pictures attributable to category I (2/42). The different morphologies, shown by the different distributions of categories, are a further indication that the two cultures differ from each other.

#### 2.3.5. Phenotypical Resistance of LOPs to Antimycobacterial Compounds

**Figure 9:**
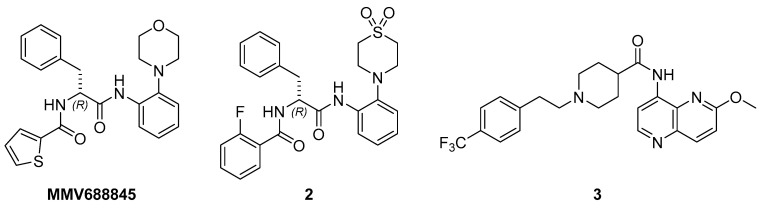
Experimental antimycobacterial compounds: MMV688845 and compound **2** belong to the class of AAPs, which are RNA-polymerase inhibitors [45,46], and compound **3** is a new DNA gyrase inhibitor [47].

The most relevant attribute of mycobacterial NRPs is their robustness against a variety of antimycobacterial compounds. This is well proven for *M. tuberculosis* [40,41,42] and one of the reasons for the long treatment duration. Yam and colleagues presented a study on a variety of clinically relevant compounds that can be used in the treatment of infections with *M. abscessus* Bamboo, demonstrating that this also applies to *M. abscessus* subsp. *abscessus* [10]. To further ensure the LOP state of a culture subjected to our assay, the differences in the bactericidal activities of several compounds were investigated under aerobic vs. hypoxic conditions. Although there is still no standard therapy regimen for *M. abscessus* pulmonary disease [5,43], some compounds are considered indispensable for the treatment of mycobacterial infections, particularly amikacin, clarithromycin, cefoxitin, rifabutin and moxifloxacin. Additionally, the antimycobacterial cyclodepsipeptide pyridomycin [44] was tested alongside drug candidates from two different antimycobacterial classes, *viz.* two RNA polymerase inhibitors of the *N-*α-aroyl-*N*-aryl-phenylalanine amide (AAP) class of compounds [45] (MMV688845 and compound **2** [46]) and a DNA gyrase inhibitor (compound **3** [47]). All compounds except pyridomycin were reported to show promising in vitro activity against *M. abscessus* under aerobic conditions (Figure 9).

**Figure 10:**
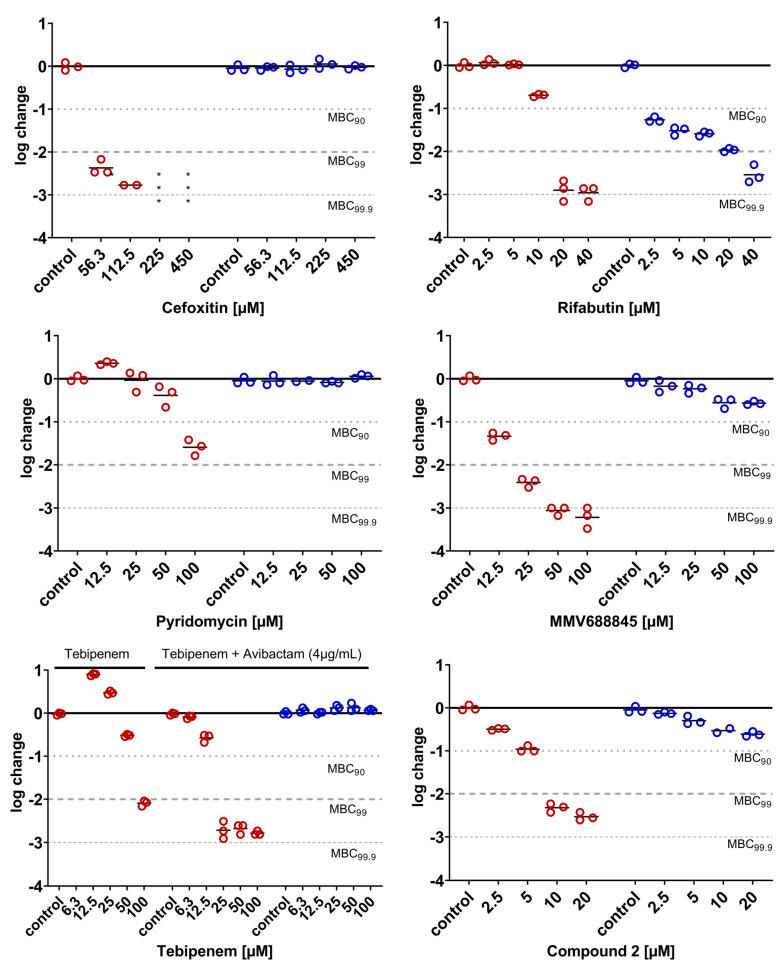
Cell wall synthesis inhibitors and RNA-polymerase inhibitors. Dose-dependent logarithmic change in CFU/mL on day 3 under aerobic (red) and hypoxic (blue) conditions. The lines represent the means of a technical triplicate, and individual values are shown as circles. The results are exemplary for at least one of two independent experiments. For the determination of activity under hypoxia, the bacterial culture was incubated under hypoxia for 6 days before treatment start. Number of * equals number of samples below LOD (100 CFU/mL).

**Figure 11:**
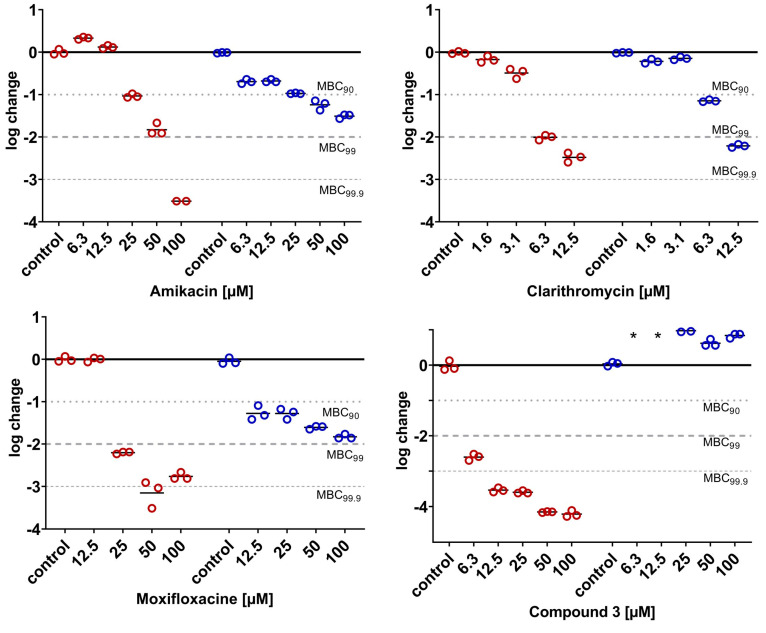
Protein synthesis inhibitors amikacin and clarithromycin and gyrase inhibitors moxifloxacin and compound **3**. Dose-dependent logarithmic change in CFU/mL on day 3 under aerobic (red) and hypoxic (blue) conditions. Lines represent the mean of a technical triplicate, and individual values are shown as circles. The results are exemplary for at least one of two independent experiments. For the determination of activity under hypoxia, the bacterial culture was incubated under hypoxia for 6 days before treatment start. Number of * equals number of samples below LOD (100 CFU/mL).

The dose-dependent activities under both conditions are displayed in Figure 10 and Figure 11. The experimental protocols are reported in the Section 3.5 and Section 3.6. The bacterial population was treated for 3 days with the respective compounds, under either aerobic or hypoxic conditions. All compounds were bactericidal to replicating *M. abscessus* (Figure 10 and Figure 11, aerobic conditions, red circles), reducing the CFU values by at least 2 log units, with the exception of pyridomycin, which led to a 1.5-log reduction only at no less than 100 µM. When comparing the activity under aerobic conditions with the activity on LOP cells determined under hypoxic conditions (blue circles, Figure 10 and Figure 11), for most compounds, a loss of bactericidal activity was found. According to their behaviors under hypoxic conditions, the compounds can be categorized into four groups: (I) compounds that showed no reduction in CFUs at all under hypoxic conditions (cefoxitin, pyridomycin, tebipenem (even though the lactamase inhibitor avibactam was added) and compound **3**), (II) compounds that reduced CFUs but failed to reach a reduction of 90% (MMV688845 and compound **2**), (III) compounds that were able to reduce 90% of CFUs under hypoxic conditions (amikacin and moxifloxacin)) and (IV) those that reduced 99% of CFUs under the investigated concentrations (rifabutin and clarithromycin).

Since all compounds showed lower activity under hypoxia than under aerobic conditions, the results are in line with our expectations. The inactivity of targets due to their silencing as a response to hypoxic stress is a possible reason for the reduced activity. This is supported by the significant changes in the proteome under hypoxic compared to aerobic conditions. For an overview of the literature on *M. tuberculosis’* expression of dormancy genes and metabolic shift, see [24]; for the literature on *M. abscessus,* see [21,23,48]. LOPs replicate markedly less. This should result in their lower sensitivity to compounds that target cell wall synthesis [15]. The complete loss of bactericidal activity against LOPs seen here in inhibitors of cell wall synthesis (Figure 10) is consistent with findings in the literature [10,15]. Another reason for the loss in activity could be the lower penetration rates under hypoxic conditions due to the altered cell walls of bacteria in response to hypoxic stress. For *M. tuberculosis*, the morphological changes in the membranes of NRPs compared to actively dividing cells were investigated by atomic force microscopy (AFM) and scanning electron microscopy (SEM) by Jakkala et al. in 2019. They described a significantly thicker outer layer, although the peptidoglycan and electron transparent layer were unchanged [49]. This fact impedes the further permeation of drugs through the thick lipophilic cell wall, and is thought to be a reason for the reduced activity of drugs against *M. tuberculosis* NRP.

Table 1 summarizes the MIC and MBC values determined in this study, and compares them with reported values. The clinical importance of non-replicating persisters is still underestimated. For instance, there are only two studies [10,22] that have reported their activities on the respective compounds under hypoxic conditions, although some of the compounds are routinely administered in the treatment of *M. abscessus* infections. In contrast to the results in the literature, we provide data covering a range of concentration of the drugs. Therefore, our study can help us to understand the antimicrobial efficacy of clinically relevant compounds.

Table 1 shows that the MICs determined in our study are largely consistent with the results in the literature. Differences in the treatment length, model setup and strains used between the literature and this study complicate the direct comparison of MBC values. Although rifabutin is one of the most important drugs used in the treatment of mycobacterial infections, Lanny et al. are the only ones to have previously reported on its activity under hypoxic conditions. Our results confirm their finding of higher activity under hypoxia compared to aerobic conditions at low concentrations. However, at higher concentrations, slightly lower activities were determined under hypoxia. Nevertheless, in our study, this drug was the most active one under hypoxia among those investigated. This finding suggests the potential importance of rifabutin in the treatment of *M. abscessus* infections.

The two drug candidates of the compound class of AAPs showed different patterns in their activity shifts, acting on the same target as rifabutin but binding to a different target site [50]. MMV688845 and compound **2** showed >2 log reduction at 25 µM and 10 µM, respectively, but reduced *M. abscessus* LOPs by 0.5 log at 50 µM and 10 µM only. At a first glance, this result is surprising, because rifabutin’s activity in hypoxia proves that the target is still active. At the moment, it can only be assumed that different permeation rates caused this.

Clarithromycin, a very important drug used in the clinic, retained its activity under hypoxia (Figure 11, bottom middle). There are conflicting reports on the bactericidal activity of clarithromycin in replicating *M. abscessus*. Several groups found bactericidal activity [22,51,52] reaching as high as a 3-log reduction within three days at 21 µM [51], while others found a bacteriostatic effect only [10]. The differences under aerobic conditions between Yams and our results are consistent with the differences under hypoxia, probably due to the differences in susceptibility to aminoglycosides between the strains. However, our results confirm the shift in activity under hypoxic conditions as observed by Lanni et al. [22].

Our results for amikacin confirm the finding of Kolpen et al., who reported an increase in the killing of oxygenated *M. abscessus* cultures at high concentrations of amikacin compared to anaerobic ones [53]. Our results are again different to Yam’s results, as they could not determine any bactericidal activity under hypoxia, further supporting a difference in the susceptibility of the strains. However, our results confirm that this mainstay of therapy has very low activity towards *M. abscessus* LOPs.

Moxifloxacin showed a bimodal shift of activity comparable to that of rifabutin—a higher activity under hypoxia at low concentrations, and a lower activity under hypoxia at higher concentrations. Our results are in the same range as Lanni’s, who reported an MBC_90_ > 10 µM under hypoxia, but they are again contrary to Yam’s results, who did not find any killing at all up to 100 µM [10,22].

The multiple changes in the phenotypical drug susceptibility of *M. abscessus* towards a variety of drugs from different compound classes support the presupposition that culturing *M. abscessus* in the described setup indeed leads to the formation of LOPs from a replicating culture. At the same time, these results confirm alarming reports that there are almost no drugs available in the clinic that are able to eradicate this critical bacterial subpopulation.

**Table 1:** MIC_90_ and MBC values determined under aerobic and hypoxic conditions in comparison to results in the literature obtained under the hypoxic Wayne model.

				Literature Data			
Drug	MIC_90_ (µM) Aer	MBC (µM) Aer	MBC (µM) LOPs	MIC_90_ (µM) Aer	MBC (µM) Aer	MBC (µM) Wayne	Ref.
Pyridomycin	12.5	MBC_90_ 100	MBC_90_ n.a.	11.6	-	-	[44]
		MBC_99_ n.a.	MBC_99_ n.a.				
Cefoxitin	28	MBC_90_ 56	MBC_90_ n.a.	16	MBC_90_ 25 ^a^	MBC_90_ > 100	[10]
Tebipenem (+Avibactam 4 µg/mL)	6.3	MBC_90_ 25	MBC_90_ n.a.	3–4	MBC_90_ 8	-	[52]
		MBC_99_ 25	MBC_99_ n.a.		MBC_99_ 16	-	[52]
					MBC_99.9_ 24	-	[52]
Rifabutin	1.3	MBC_90_ 20	MBC_90_ 2.5	2.3	MBC_90_ > 3.5 ^b^	MBC_90_ 3.5 ^b^	[22]
Clarithromycin	0.8	MBC_90_ 6	MBC_90_ 6.3	0.2	MBC_90_ > 100 ^a^	MBC_90_ > 100 ^a^	[10]
		MBC_99_ 6	MBC_99_ 12.5	0.3	MBC_99_ 2.7 ^b^	MBC_90_ 2.7 ^b^	[22]
Amikacin	6.3	MBC_90_ 25	MBC_90_ 25	2	MBC_90_ 12.5 ^a^	MBC_90_ > 100 ^a^	[10]
				13.6	MBC_90_ > 13.6 ^b^	MBC_90_ > 13.6 ^b^	[22]
Moxifloxacin	6.3	MBC_90_ 25	MBC_90_ 12.5	2	MBC_90_ 3 ^a^	MBC_90_ > 100 ^a^	[10]
		MBC_99_ 25	MBC_99_ n.a.	10	MBC_99.9_ 10 ^b^	MBC_90_ > 10 ^b^	[22]

Aer: aerobic incubation. LOP values were determined via the developed method. n.a.: not achieved. ^a^ 2 days; ^b^ 7 days.

## 3. Materials and Methods

### 3.1. Source of the Materials

The reagents were purchased and used as received. Sigma Aldrich, St. Louis, MO, USA): Middlebrook 7H10 agar, Middlebrook 7H9 broth base, catalase (from bovine liver), polysorbate 80. Pan Reac AppliChem GmbH, Darmstadt, Germany: oleic acid (Ph. Eur. grade). Roche Diagnostics: bovine serum albumin. Grüssing, Filsum, Germany: glucose, glycerol (99%, water free) Na_2_CO_3_ (99.5%, anhydrous), NaHCO_3_ (99%). ORG Laborchemie, Bunde, Germany: NaCl (99.5% purity). Local hardware store: iron wool (6.5–10 g grade 0). Cell Signaling Technology, Danvers, MA, USA: mounting medium (Signal Stain^®^).

Antibacterial compounds were purchased as follows and dissolved in DMSO (unless otherwise stated): amikacin, 98% (Alfa Aesar, Haverhill, MA, USA dissolved in water and subsequently sterile filtered); avibactam, >98% (BLDpharm (BLD), Shanghai, China); bedaquiline, 99% (AmBeed, Arlington Heights, Illinois, USA); cefoxitin, >98% (Tokyo Chemical Industry Germany (TCI-Germany), Eschborn, Germany); clarithromycin, >98% (TCI Germany); metronidazole, 99% and niclosamide, 97% (VEB Sächsisches Serumwerk, Dresden, Germany); moxifloxacin, >98% (Sigma Aldrich, St. Louis, MO, USA); pyridomycin (kindly provided by Prof. Dr. Shuangjun Lin, State Key Laboratory of Microbial Metabolism, Joint International Laboratory on Metabolic & Developmental Sciences, School of Life Sciences & Biotechnology, Shanghai Jiao, Tong University, Shanghai, China, analyzed by NMR before use), rifabutin, >98% (TCI Germany); tebipenem, 98% (BLD). The 3-hydroxychromone dye trehalose conjugate (compound **1**) was synthesized as described in reference [54]. Compound **2**, MMV688845 and **3** (dissolved in ethanol) were synthesized as described in the literature [46,47].

Consumables were purchased from the following companies: Saarstedt (Nümbrecht, Germany)—single-use inoculation loop, 50 mL tubes, 2 mL screw cap tubes, 96-well plates (83.3924.500); Epredia (Portsmouth, NH, USA)—microscope slides (25 × 75 × 1 mm); Brand (Sigma Aldrich, St. Louis, MO, USA)—square glass cover slips.

### 3.2. Bacterial Strain and Culture Medium (Liquid and Solid Medium)

Preparation of working stocks: *M. abscessus* subsp. *abscessus* ATCC 19977 (rough morphotype) was spread on solid medium (a freshly prepared Middlebrook 7H10 agar plate supplemented with 0.2% oleic acid), and 5% bovine serum albumin, 0.2% glucose, 0.0004% catalase, 0.08% NaCl and 0.05% glycerol were added with a sterile single-use inoculation loop and incubated for 3 days at 37 °C, 5% CO_2_. Then, 15 mL of liquid culture medium (Middlebrook 7H9 broth supplemented with 0.08% NaCl, 0.2% glucose, 5% bovine serum albumin and 0.05% polysorbate 80) in a 50 mL tube was inoculated by picking a single colony from a fresh streak plate. After 3 days of incubation at 37 °C (5% CO_2_), the culture was diluted and further incubated until the OD_600_ reached 0.6–0.8. The culture was aliquoted by adding 500 µL of the culture into screw cap tubes containing 500 µL of 50% glycerol (in 7H9 broth base, final concentration of glycerine 25%). The screw cap tubes were stored at −80 °C until further use.

### 3.3. Minimum Inhibitory Concentration (MIC) Determination in 7H9 by OD_600_ Measurement

**Figure 12:**
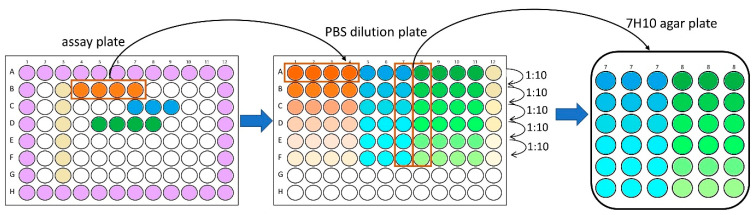
Graphical illustration of MBC determination procedure by PBS dilution preparation and spot plating on solid medium. The procedure shows three compounds, selected for MBC determination on solid medium (orange, blue and green wells of assay plate) and the subsequent preparation of a 10-fold serial dilution of these wells in another 96-well plate (PBS dilution plate). Spot plating on square 7H10 agar plates was shown to be exemplarily for two wells only (column 7 and 8 of the PBS dilution plate) in triplicate. Purple wells in the assay plate contain medium only in order to reduce the evaporation of corner wells, while the wells in column 3 (pale yellow) contain the negative control used to determine 100% growth.

Here, 10 mL of liquid medium was inoculated by transferring 1 mL of a working stock into a 50 mL tube and incubating at 37 °C for 24 h. Thereafter, the culture was sub-cultured once and further incubated to reach a mid-log phase with an OD_600_ between 0.4 and 0.8, where an OD_600_ of 0.5 corresponds to 5 × 10^8^ CFU/mL. Then, the required volume of liquid culture medium (see preparation of working stock) was inoculated to reach about 5 × 10^5^ CFU/mL, while some experiments were also performed with 5 × 10^7^ CFU/mL. MICs were determined by performing a serial dilution broth assay in 96-well plates with a total volume of 200 µL in each well. To reduce evaporation effects, compounds and references were used, starting from column two and row B, respectively, and the wells at the borders of the plate were filled with 200 µL medium. For a schematic depiction of the plate layout, see Figure 12. Column two contained 5 µM bedaquiline as a positive control for antimycobacterial activity, and column three contained 1% DMSO as an untreated control. All compounds were used as a stock solution at 100 times the highest concentration of the intended initial concentration. Then, 4 µL samples of the stock solutions were transferred to the wells of column four, which contained 200 µL of pure medium. Subsequently, the wells were serially diluted using a multichannel pipette (Eppendorf). Thereafter, 100 µL of the prepared inoculum was distributed into each well. The plate was incubated at 37 °C in 5% CO_2_ for three days and growth inhibition was determined by OD_600_ measurement (FLUOstar OPTIMA, BMG Labtech, λ = 590 nm).

### 3.4. Calculation of MIC_90_

MIC_90_ was used to determine the activity under aerobic conditions in order to select the right wells for MBC determination. Each assay plate contained six wells of 1% DMSO and six wells of 5 µM bedaquiline as controls. Raw OD values were used to calculate the percentage of growth inhibition compared to DMSO and bedaquiline, according to the following formula:

(1) $$\frac{OD\;(sample)-mean\;OD\;(bedaquiline)}{mean\;OD\;(bedaquiline)-mean\;OD\;(DMSO\;control)}\times \left(-100\right)=\%inhibition$$

The lowest concentration exceeding 90% inhibition is reported as MIC_90_.

### 3.5. LOPs Assay Under Hypoxic Conditions

A box equipped with a tightly sealable lid was used as a hypoxic chamber. Of note, anaerobic pots with a volume of 2.5 L usually used for agar plates were also successfully used as the hypoxic chamber (picture of a setup with an anaerobic pot in Supplementary Materials Figure S1). We used a box with 2.5 L volume. The reduction in oxygen content in a closed system followed a published method, using the conditions described as most effective [25]. In brief, 7 g iron wool was freshly activated as described in [25] with a CuSO_4_ solution (1%) containing 0.5% polysorbate 80 as the wetting agent by submerging the iron wool into the solution for 30 s. The pH of the solution was adjusted to 1.5 with concentrated sulfuric acid. Stabilized methylene blue (MB) solution [25] was used as an indicator for the onset of hypoxia and a saturated carbonate solution (Na_2_CO_3_/NaHCO_3_, 1:1) served as the CO_2_ source. Both solutions were placed inside the chamber with a 96-well plate filled with the inoculum. Subsequently, the lid was tightly closed (schematic depiction of the setup shown in Figure 3; for a photo of the setup in our laboratory, see Supplementary Materials Figure S2). The box was incubated at 37 °C for six days to ensure transition into LOPs. Thereafter, serial dilutions of compounds were prepared in a new 96 well plate (compound plate) yielding a total volume of 140 µL. After opening the hypoxic chamber, 100 µL of each well of the compound plate was quickly transferred to the hypoxic inoculum plate and the iron wool was replaced with a freshly activated piece. The box was closed again and further incubated at 37 °C for three days. To investigate the activity of metronidazole and niclosamide, incubation was performed for six days in some experiments. Thereafter, the steps described in the section on MBC determination were carried out to determine compound activity.

### 3.6. MBC Determination on Agar

After the incubation of a microtiter plate for the desired time under either aerobic or hypoxic conditions, wells were selected for MBC determination. For a compound that showed activity against *M. abscessus* under aerobic conditions, four to six concentrations were chosen, with the MIC being the lowest of these concentrations. If the assay was performed under hypoxic conditions, the concentrations were selected according to results derived under aerobic conditions to compare results. The selected wells of the assay plate were resuspended using a manual multichannel pipette (~20 cycles) and transferred to row A of a new 96-well plate (PBS dilution plate). Tenfold serial dilutions of each sample were prepared using PBS (Carl Roth) supplemented with 0.025% polysorbate 80. Subsequently, six wells of each column (six dilutions of each sample, 10 µL each) were spot plated in triplicate on solid medium (see preparation of working stocks) containing 0.4% activated carbon to prevent the possible inhibitory effect of the transferred (active) compound, using a multichannel pipette (Move it^®^-Eppendorf). The plates were incubated at 37 °C in an atmosphere containing 5% of CO_2_. After the colonies reached a countable size (usually 2–3 days for *M. abscessus*, depending on the impact of hypoxia and the post-antibiotic effects of the investigated compound), pictures of each spot were taken with a digital microscope (Tomlov DM602 Pro 10.1 Inch HDMI, Shenzhen, China). Colonies within one spot were counted using the software DotDotGoose version 1.7.0 [55]. Dilutions with clearly distinguishable colonies closest to 50 were chosen for the calculation of CFU/mL for all samples of interest. The MBC_90_ was defined as the lowest compound concentration that exceeded 90% reduction in CFU/mL.

### 3.7. Calculation of Log Change

We calculated the logarithmic change compared to the inoculum according to the following formula:

(2) $$log\;change={log}_{10}\left(\frac{CFU}{mL}(sample)\right){-log}_{10}\left(mean\frac{CFU}{mL}(drug\;free\;control)\right)$$

The CFU/mL in the drug-free control was determined in aerobic samples on the day when compounds were added to the bacterial culture (inoculum) and in hypoxic samples at the end of hypoxic treatment, to exclude any change in CFU/mL caused by hypoxia alone.

### 3.8. Fluorescent Labeling of M. abscessus Cells and Preparation of Samples for Microscopy

The culture of interest (aerobic or hypoxic) was stained in either 96-well plates or in 2 mL screw cap tubes with 100 µM of compound **1** (10 mM in DMSO), manually mixed and subsequently incubated for 2.5 h at 37 °C. When staining was performed in 96-well plates, the stained cultures were afterwards combined in a 2 mL tube. The cultures were pelleted at 6500 rpm for 7 min. The supernatant was replaced with the same volume of a 4% paraformaldehyde (PFA) solution in PBS (Carl Roth) and fixed for 30 min. The sample was washed twice with PBS by pelleting at 6500 rpm for 7 min. After the last washing step, the supernatant was removed as far as possible to reduce background signals of the dye. Then, 50 µL of ethanol (100%) was added and homogenized. Then, 10 µL of the suspension was transferred onto conventional microscope slides drop by drop, allowing the evaporation of ethanol before the next drop was added on the same spot. Then, 10 µL of mounting medium was added on top and covered with square glass coverslips. A negative control was prepared containing unstained aerobic *M. abscessus* cells. The samples were allowed to dry overnight and investigated in a single-blinded study with confocal laser scanning microscopy.

### 3.9. Confocal Laser Scanning Microscopy

Images of *M. abscessus* were acquired using a confocal laser scanning microscope (Nikon A1R) equipped with a 60× oil immersion objective (numerical aperture = 1.4, Plan Apo Lambda, Nikon, Tokyo, Japan). The green fluorescence of compound **1** was excited using the 488 nm laser line of an argon laser (Melles Griot, Rochester, MN, USA) and fluorescence emission was detected in the spectral range of 500–550 nm using a photon multiplier detector unit (Nikon, Tokyo, Japan). Confocal images were acquired at an additional 8× digital zoom. Laser power and detector gain levels were set such that bacterial structures were clearly visible without driving the detector into saturation. The images were processed using NIS-Elements software (Nikon, Tokyo, Japan).

### 3.10. Automated Object Counting Using Fluorescence Microscopy

The procedure was adjusted from a published method [37]. Briefly, bacterial cultures in wells of 96-plates incubated under aerobic or hypoxic conditions were mixed using a manual multichannel pipette, and 2 µL of each well was transferred to a plate containing 198 µL of fresh liquid medium. These plates were incubated in 37 °C (5% CO_2_) to allow the regrowth of cells. At different points in time, 2 µL of compound **1** (10 mM in DMSO) was added to the bacterial suspension to stain the cells and allowed to incubate for another 2.5 h. Thereafter, the wells were homogenized, and 1 µL of the suspension was transferred to a black-walled, clear, flat-bottom 96-well plate containing 199 µL of filtered 4% PFA solution in PBS for fixation (dilution step due to background noise of the dye). Fluorescence microscopy was then performed using a Thermo Fisher Scientific CellInsight CX5 instrument (Waltham, MA, USA). The samples were analyzed at λ_ex_ = 485 nm and λ_em_ = 510–531 nm. Then, 21 independent images were acquired per well and analyzed. The valid object count of the GFP filter was matched to the number of bacteria counted. The valid field count was used to check that all fields were in focus under the microscope.

### 3.11. Measuring of CFU Characteristics (Area, Perimeter and Length) on Pictures

Pictures of spots with several colonies were taken with a digital microscope (see Section 3.6) and analyzed using a free copy of Digimizer Version 6.4.3 (MedCalc Software Ltd., Ostend, Belgium). For this, pictures were loaded into the software and processed by binarization. Threshold levels were adjusted to ensure the detection of colonies as objects. The statistical analysis of the obtained values was performed using Graph Pad Prism.

### 3.12. Oxygen Measurement

Oxygen concentration was measured with an optical microsensor (NTH-PSt1 connected to Microx TX3 Presens Precision Sensing GmbH, Regensburg, Germany). The instrument was calibrated immediately before the measurement of the sample following the instructions in the user manual given by the manufacturer. The values are reported as relative percentages of oxygen compared to air-saturated water that has, by definition, an oxygen saturation of 100%. The conversion of values into other units was performed using a calculation software that was provided by the manufacturer of the measurement device, while the recorded temperature was included in the calculation.

## 4. Conclusions

We have here developed a new model to investigate *M. abscessus* under hypoxic conditions in an easy, safe and cost-efficient way. Compound addition in a microtiter plate format after culture transition into LOPs is possible under our model, in contrast to the hypoxic model using microtiter plates, where compounds need to be added before the onset of hypoxia. Our setup includes both a control for reduced oxygen content (MB) and a verification compound (either 100 µM metronidazole or 50 µM niclosamide) to ensure the transition of the culture into LOPs. The results regarding growth behavior, the morphology of the cells and the phenotypical drug susceptibility of the LOP culture demonstrate that our model leads to the transition of a log-phase culture of *M. abscessus* subsp. *abscessus,* distributed in a 96-well plate, to *M. abscessus* LOPs. Using this model, we have confirmed the well-described phenomenon that hypoxic non-replicating persisters are non-susceptible to a variety of clinically relevant drugs, including important drugs such as amikacin and cefoxitin. Studying drugs in the described LOPs model will help to understand their efficacy in the clinic. Both the Wayne model and ours are not routinely used in the clinic. The Wayne model takes too much time in a therapeutic setting. Our model is characterized by features that render it amenable to a routine diagnostic setting, such as its easier and faster setup, faster throughput of more samples, and automated evaluation of the assays.

## Data Availability

Data will be made available on request.

## Supplementary Materials

The following supporting information can be downloaded at: https://www.mdpi.com/article/10.3390/antibiotics14030299/s1, Figure S1: Hypoxic chamber for the investigation on *M. abscessus* LOPs in an anaerobic pot, commonly used in culturing anaerobic bacteria on solid medium; Figure S2: Setup for *M. abscessus* LOP assay in our laboratory, Figure S3: Determined oxygen levels, Figure S4: Representative selection of *M. abscessus* cells pre-treated under two different conditions.

## Abbreviations

The following abbreviations are used in this manuscript:

LOP	Low-oxygen persister
MB	Methylene blue
MBC	Minimum bactericidal concentration
MIC	Minimum inhibitory concentration
NRP	Non-replicating persister
OD	Optical density
