# Supplementary material for: A Simple In Vitro Method to Determine Bactericidal Activity Against Mycobacterium abscessus Under Hypoxic Conditions
Source: Antibiotics (Basel). 2025 Mar 13;14(3):299. doi: 10.3390/antibiotics14030299 (PMC11939544; doi:10.3390/antibiotics14030299)
Supplement: Supplementary file 1 [file antibiotics-14-00299-s001.zip › antibiotics-3485151-supplementary.pdf]

A simple *in vitro* method to determine bactericidal activity against *Mycobacterium abscessus* under hypoxic conditions

Supplementary Information

Ruth Feilcke, Robert Eckenstaler, Markus Lang, Adrian Richter[1], Peter Imming[2]

Institut für Pharmazie, Martin-Luther-Universität Halle-Wittenberg, Wolfgang-Langenbeck-Straße 4 06120 Halle

[1]corresponding author adrian.richter@pharmazie.uni-halle.de

[2]corresponding author peter.imming@pharmazie.uni-halle.de

Table of content

Figure S1: Picture of *M. abscessus* LOP assay set up in a common anaerobic pot.

Figure S2: Picture of *M. abscessus* LOP assay set up in hypoxic box in our laboratory.

Figure S3: Oxygen levels in aerobe medium and different parts of the hypoxic set up.

Figure S4: Representative selection of *M. abscessus* cells incubated under aerobe (A) and hypoxic (B) conditions.

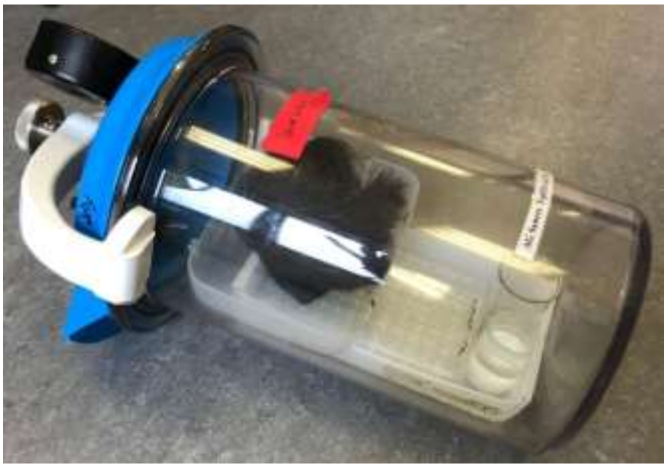

Figure S 1: Hypoxic chamber for the investigation on M. abscessus LOPs in an anaerobic pot, commonly used in culturing anaerobic bacteria on solid medium. Due to the round shape, the set up needs to be performed in a lying position and vials with indicator (top, right side) and carbonate solution (bottom, right side) are placed in a tray to avoid tipping over. The activated iron wool is placed in an extra tray (to prevent contamination by oxidation products) on top of the microtiter plates. Due to the unfavourable shape, this set up takes up a lot of space, but it can be used if no other airtight boxes are available.

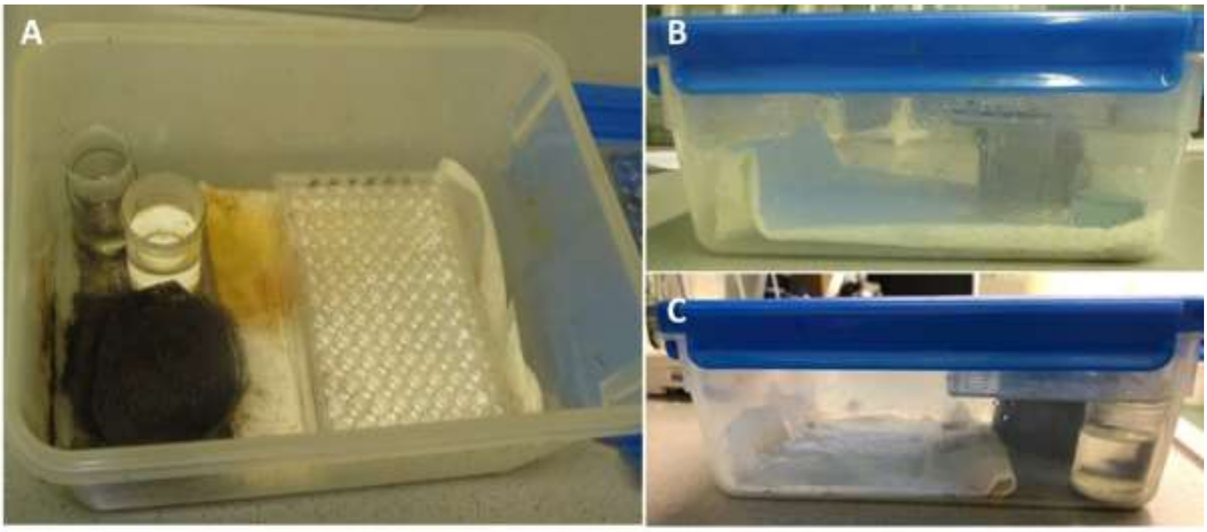

*Figure S 2: Set up for M. abscessus LOP assay in our laboratory. A square plastic box with a "lock-n-lock" lid (equipped with rubber to ensure airtight sealing) is used as hypoxic chamber. (**A**) A microtiter plate, a glass vial with stabilized MB solution (top left), a glass vial with saturated carbonate solution (middle left) and activated iron wool (bottom left), placed in a crystallizing dish to hold the access copper sulphate solution, are placed next to each other in the box. If several plates are investigated at once, these can be stacked on each other. (**B**) Hypoxic chamber immediately after closing (MB still blue). (**C**) Hypoxic chamber after onset of hypoxic conditions (MB decolorized). The lid on top of the glass vials and the iron wool is a protection to prevent contamination of the microtiter plates by the oxidation products of the activated iron wool, in case of plates being stacked on top of the vials as well. A maximum of 6 microtiter plates can be examined simultaneously.*

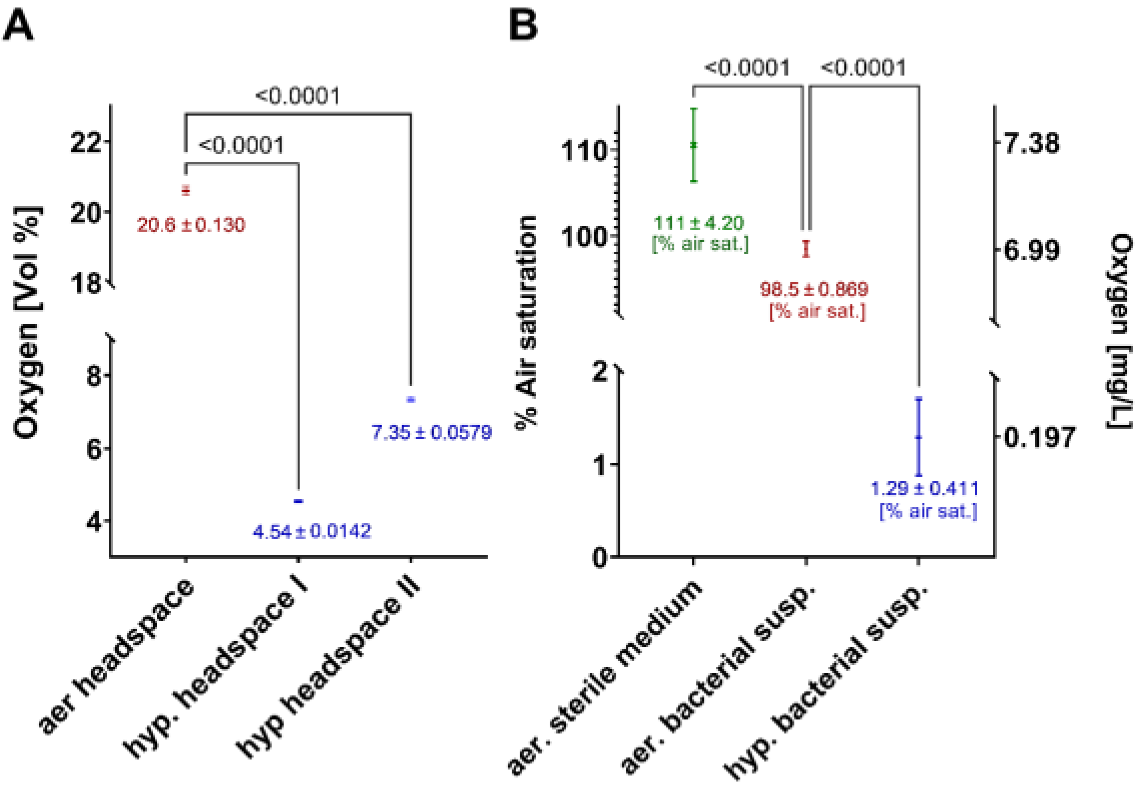

*Figure S 3: Determined oxygen levels in (**A**) headspace [Vol%] and (**B**) culture medium [% air saturation], measured each under aerobic and hypoxic conditions. Stabilized methylene blue solution was employed to survey oxygen conditions before measuring. In graph B, oxygen [mg/L] is displayed together with % air saturation to enable comparison with literature studies. These values were calculated using a calculation sheet free of charge of the manufacturer of the oxygen measurement equipment (available at: https://www.presens.de/support-services/download-center/tools-utilities - Oxygen Unit Calculation (Excel Sheet). The recorded temperatures during measurements were included in the calculation.*

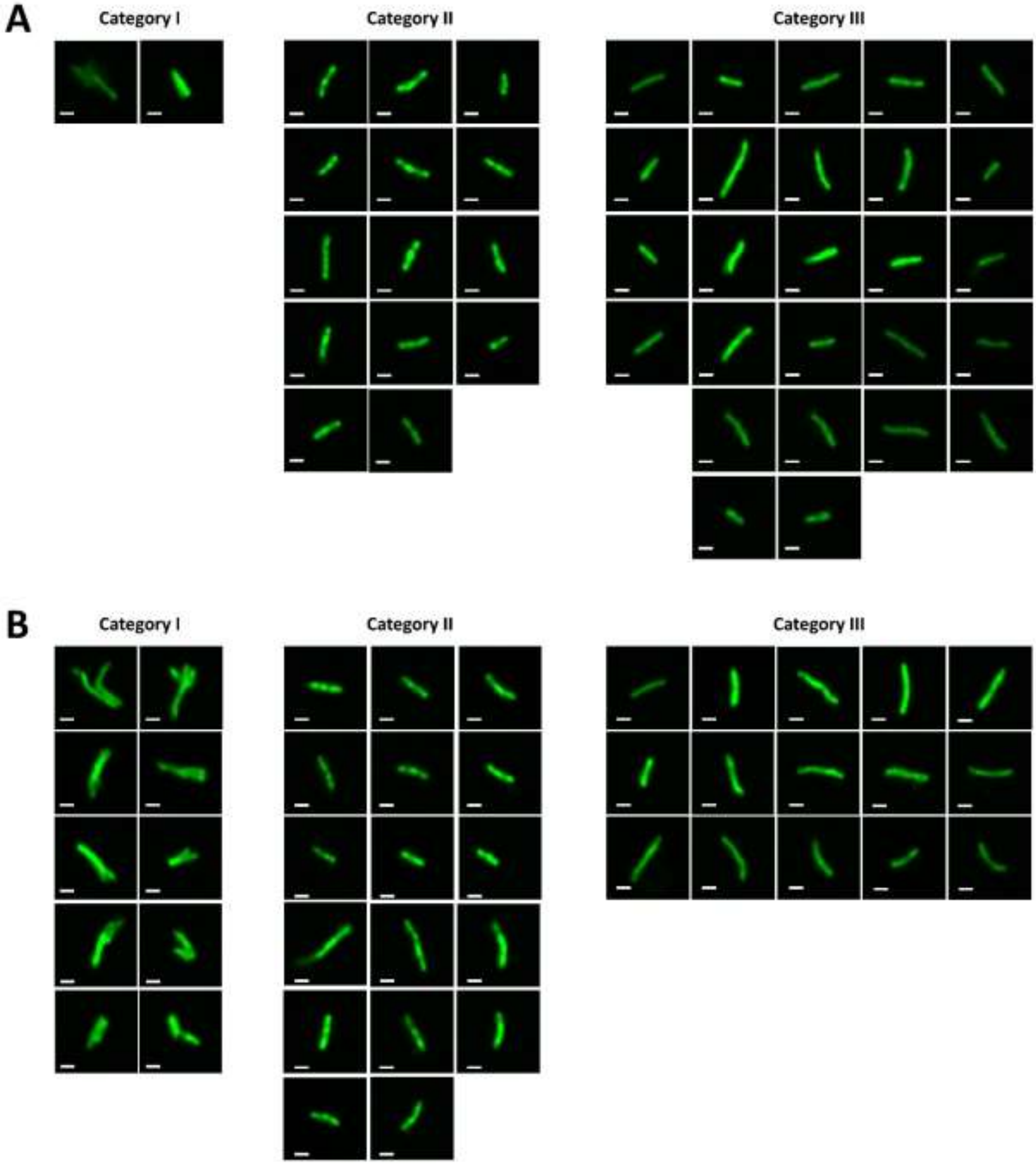

*Figure S 4: Representative selection of M. abscessus cells pre-treated under two different conditions that were afterwards stained with compound **1** and analyzed by confocal laser scanning microscopy. Pictures are sorted by their morphological appearance into three different categories as explained within the publication. (**A**) Cells from LOP-culture incubated in hypoxic box and (**B**) cells from aerobic culture incubated in 5 % CO_2, scale bar 1 µm.*
